# Management of sore throat in Danish general practices

**DOI:** 10.1186/s12875-019-0970-3

**Published:** 2019-06-01

**Authors:** Kasper Basse Reinholdt, Maria Rusan, Pernille Rosbjerg Hansen, Tejs Ehlers Klug

**Affiliations:** 1 Department of Otorhinolaryngology, Head and Neck Surgery, Aarhus University Hospital, Palle Juul-Jensens Boulevard 99, DK-8200 Aarhus N, Denmark; 2 Department of Clinical Medicine, Aarhus University, Aarhus, Denmark; 3 Department of Otorhinolaryngology, Region Hospital Holstebro, Holstebro, Denmark; 4 General practice, Doctors Rosbjerg & Henriksen, Aarhus, Denmark

**Keywords:** Sore throat, Acute pharyngitis, Guideline, Family practice, General practice, Antibiotics, Denmark

## Abstract

**Background:**

The national guideline for sore throat, endorsed by the Danish Society of General Medicine, recommends the use of the modified Centor score and streptococcal rapid antigen detection test to guide diagnosis and treatment of sore throat. The aim was to investigate Danish general practitioners (GPs) routine management of sore throat patients with a focus on the modalities used and adherence to the guideline.

**Methods:**

A cross-sectional study. GPs in the Central Denmark Region answered an online questionnaire in October 2017. The main outcome measure was modalities used in the management of sore throat patients.

**Results:**

In total, 266 of 500 (53%) GPs answered the survey. Ten percent of participants were adherent or almost adherent to the guideline, while 82% of GPs added one or more extra modalities (general clinical assessment (67%), biochemical parameters (48%), and throat swabs for bacterial culture (18%)) to differentiate viral and bacterial etiology. Sixty-five percent of participants used the Centor Score or modified Centor Score, 96% of GPs used a streptococcal rapid antigen detection test, and all GPs chose narrow-spectrum penicillin as the first-line antibiotic. The most common reasons for non-adherence to the guideline were greater confidence in the clinical assessment (39%), time pressure (33%), and difficulty recalling the guideline (19%).

**Conclusion:**

Danish GPs rarely adhere to the recommended sore throat management guideline, but use various combinations of different modalities in the assessment of bacterial infection. This practice may increase antibiotic prescription rates.

**Electronic supplementary material:**

The online version of this article (10.1186/s12875-019-0970-3) contains supplementary material, which is available to authorized users.

## Background

Acute pharyngo-tonsillitis (sore throat) is a common reason for consulting general practitioners (GPs) [1]. The infection is usually self-limiting and 70% of cases are assumed to be viral in etiology, while the remaining are caused by bacterial pathogens [1–3]. The current Danish Society of General Medicine guideline for sore throat focuses on the detection of Group A streptococci (GAS) and limiting unnecessary antimicrobial therapy (Table 1). The guideline is based on the modified Centor Score (McIsaac Score) to estimate the probability of GAS infection and, thus, guide GPs in the use of the streptococcal rapid antigen detection test (RADT) and the prescription of antibiotics [4].

**Table 1:** Guideline for the management of patients with acute pharyngo-tonsillitis^a^

Modified Centor Score	Guideline
0–1	No streptococcal RADT, no antibiotic treatment.
2–3	If streptococcal RADT is positive treat with antibiotics.
4–5	If streptococcal RADT is positive treat with antibiotics or if patient is generally unwell treat with antibiotics without performing streptococcal RADT.

^a^Respiratory tract infections - diagnosis and treatment 2014 (Danish Society of General Practitioners, DSAM). http://vejledninger.dsam.dk/luftvejsinfektioner/ Accessed 19 July 2018

*Abbreviation*: *RADT* Rapid antigen detection test

To calculate the modified Centor score, patients receive 1 point for each of the following symptoms and findings: Anamnestic fever, absence of cough, presence of tonsillar exudates, and tender cervical lymph nodes. In addition: Age < 3 years: − 1 point. Age 3–14 years: 1 point. Age 15–44 years: 0 points. Age > 45 years: − 1 point

Previous studies found that antibiotics were prescribed to 45–70% of Danish sore throat patients [5–7] and similar results have been reported from other countries, such as USA (47%) [8], Spain (74%) [9], and Belgium (76–84%) [10]. While previous quantitative studies on adherence to sore throat guidelines focus on the modalities used to select patients for antibiotic treatment and / or the choices of antibiotics, the current study was designed to also gain insight into how GPs deviate from the guideline and the reasons for deviation from the recommended guideline [4, 6, 11–13]. This latter information is important in devising strategies to increase guideline adherence and reduce antibiotic prescription rates. Thus, the current study aimed to investigate the routine management of sore throat by Danish GPs with a focus on exploring the modalities used and identifying reasons for not adhering to the recommended guideline.

## Methods

### Study design

We invited via e-mail 500 GPs in the Central Denmark Region to participate in this cross-sectional study. Of the 811 GPs in the Central Denmark Region we lacked email contact information for 311. An online questionnaire was developed by the authors, in collaboration with the Research Unit for General Practice at Aarhus University, Denmark.

### Data collection

Answers from the participating GPs were collected using the Research Electronic Data Capture (REDCap) system. The study was conducted between October 3rd and October 9th, 2017. Demographic data (gender and age) on all GPs in the Central Denmark Region were obtained from the Organization of General Practitioners in Denmark.

### The questionnaire

The questionnaire consisted of four parts: 1. Information regarding the participant (age, gender, number of years in general practice, additional staff involved in the management of sore throat patients). 2. Questions on the modalities used in the management of sore throat patients (RADT, clinical assessment, C-reactive protein (CRP) and / or leukocyte count, throat swab for bacterial culture, Centor Score, and modified Centor Score). 3. Questions on the criteria for antibiotic treatment and the antibiotics used. 4. Questions on the participant’s knowledge and use of the current guideline on sore throat management (see below) and reason for deviating from this. The questionnaire can be found in the appendix (see Additional file 1).

### Categorization of adherence to guideline

Answers were categorized as ‘adherent’ if they were in accordance with the Danish Society of General Medicine sore throat management guideline (referred to as ‘the guideline’) (Table 1) and if no additional modalities were used. Answers were categorized as ‘almost adherent’ if the Centor Score was used instead of the modified Centor Score (i.e. antibiotic treatment in cases with Centor Score ≥ 2 and subsequent positive RADT or patients with Centor Score of 4 that are generally unwell). Answers not complying with these criteria were considered ‘non-adherent’.

### The Danish health care system

In Denmark, patients may consult their GP free of charge. GPs receive a fee for performing a streptococcal RADT, which covers the expense of the kit, as well as a small reimbursement to the GP. Blood tests and throat swab cultures are free of charge and without additional fee for the GPs. It is standard procedure in Central Denmark Region to perform both standard culture and culture for *Fusobacterium necrophorum.* Only doctors may prescribe antibiotics to patients.

### Statistical analyses

To test the representability of the study population, the Fisher’s exact test was used to compare categorical variables (gender) and the Kruskal-Wallis test was used to compare continuous variables (age) between study participants and all GPs in the Central Denmark Region. The Fisher’s exact test was also used to compare the modalities used and reasons for nonadherence to the guideline by GPs claiming to use the guideline versus GPs admitting to not following the guideline.

### Ethical considerations

The study was approved by the Danish Data Protection Agency (2015-57-0002) and data were handled confidentially in strict accordance with the guidelines. According to Danish law, approval of the study from the local ethical committee was not required.

## Results

**Fig. 1:**
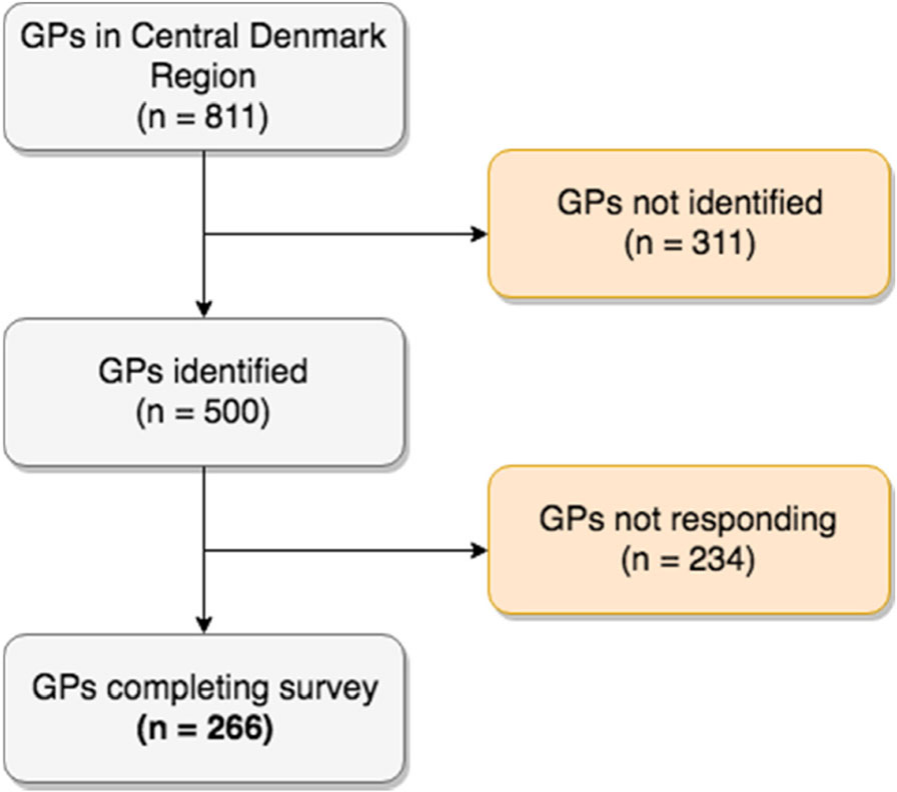
Flow chart of GP recruitment for study

In total, 266 of 500 (53%) invited GPs completed the questionnaire (Fig. 1). Participants’ age and gender distributions were similar to those of GPs in the Central Region Denmark in general (Table 2) [14].

**Table 2:** Demographics of general practitioners participating in the study and in the Central Denmark Region

Variable	Participants	Total^a^	*p*-value
	(*n* = 266)	(*n* = 811)	
*Age (years), mean*	47.0	51.7	0.175^b^
30–39 years, n (%)^+^	75 (28%)	66 (8%)	
40–49 years, n (%)	101 (38%)	293 (36%)	
50–59 years, n (%)	55 (21%)	246 (30%)	
60–69 years, n (%)	32 (12%)	196 (24%)	
≥ 70 years, n (%)	3 (1%)	10 (1%)	
*Sex, n (%)*			0.228^c^
Male	114 (43%)	384 (47%)	
Female	152 (57%)	427 (53%)	
*Years in general practice, median (range)*	5 (0–35)	NA	

^a^Data from the Organization of General Practitioners in Denmark (2017)

^b^Kruskal-Wallis test

^c^Fisher’s exact test

*Abbreviation*: *NA* Not available

^+^Of note, 75 general practitioners claimed their age was 30–39 years in the study, while the total number of general practitioners in this age group was 66 according to the Organization of General Practitioners. The reason for this discrepancy may be that data from the Organization of General Practitioners were obtained in January 2017 and the current study was conducted in October 2017

Eighty-three percent of GPs were directly involved in the management of sore throat patients, while 17% of GPs responded that sore throat patients were exclusively managed by additional practice staff. In 62% of cases, patient management was carried out partly by GPs and partly by practice staff. Eighty-three percent of practice staff used a guideline local to the practice (not specified) and 38% used the Danish Society of General Medicine guideline. Twenty-five percent claimed to use both.

### Modalities used in the management of sore throat patients

The majority of GPs used either the Centor Score (*n* = 132, 50%) or the modified Centor Score (*n* = 40, 15%) in their management of sore throat patients (Table 3).

**Table 3:** Modalities used in the management of sore throat patients by 266 Danish general practitioners

Modality	Modified Centor Score (*n* = 40)	Centor Score (*n* = 132)	No use of (modified) Centor Score (*n* = 94)	In total, n (%) (*n* = 266)
Streptococcal RADT^a^	37	125	94	256 (96%)
Clinical assessment	24	81	73	178 (67%)
CRP^b^ and/or leukocyte count	18	60	51	129 (48%)
Throat swab culture	12	25	12	49 (18%)

Note that values are number of answers in the given category

*Abbreviations*: ^a^*RADT* Rapid Antigen Detection Test. ^b^*CRP* C-reactive protein

Almost all (96%) GPs used a streptococcal RADT, but 217 (82%) of GPs added one or more additional modalities: general clinical assessment (67%), biochemical parameters (48%), and throat swabs for bacterial culture (18%). Twelve GPs (5%) used a streptococcal RADT in all patients (regardless of (modified) Centor Score).

### Criteria for antibiotic prescription

GPs used multiple criteria (i.e. modified Centor Score / Centor Score, positive RADT, general clinical assessment, throat swabs, CRP / leukocyte count) for deciding on antibiotic treatment of patients with sore throat. Only three (1%) and 23 (9%) participants were adherent or almost adherent to the guideline in regards to antibiotic prescription, respectively. Hence, 240 (90%) GPs were non-adherent to the guideline.

There were multiple reasons for non-adherence. Concerning the 172 GPs who used either the Centor Score or the modified Centor Score in their management, the majority of GPs used the scoring systems incorrectly; 55 (32%) GPs performed a streptococcal RADT (and prescribed antibiotics if the test was positive) in clinically well patients with low (0–1) Centor Scores / modified Centor Scores (Table 4). In addition, 100 (58%) GPs using the scoring systems, refrained from the use of a streptococcal RADT in some patients with Centor Score (*n* = 81) / modified Centor Score (*n* = 19) less than 4, relying only on general clinical assessment (*n* = 47), biochemical measurements (CRP and / or leukocyte count, *n* = 26), and / or bacterial culture (*n* = 27).

**Table 4:** 172 general practitioners use of the modified Centor Score (*n* = 40) or the Centor Score (*n* = 132) for deciding on whether to perform a streptococcal rapid antigen detection test (RADT) and prescribe antibiotics in well and unwell patients

	Modified Centor Score						Centor Score				
	≥0	≥1	≥2	≥3	≥4	≥5	≥0	≥1	≥2	≥3	≥4
Well											
*Positive RADT*^*a*^	4	3	24	5	1	0	20	28	58	19	0
*Regardless of RADT*	0	0	1	3	10	3	0	0	4	13	15
Unwell											
*Positive RADT*	0	0	5	2	2	0	1	5	12	3	2
*Regardless of RADT*	1	1	6	7	15	1	9	8	19	28	10

Values are number of answers provided. Answers are categorized according to the lowest number of modified Centor Scores / Centor Scores indicated for well and unwell patients, respectively, regardless of and / or following a positive RADT

Concerning the 94 GPs who did not use the Centor Score or the modified Centor Score, decision on antibiotic therapy was based on one or more of the following criteria: positive streptococcal RADT (*n* = 92), clinically unwell patients (*n* = 51), elevated CRP and / or leukocyte count (cut off values unknown) (*n* = 29), positive bacterial culture (*n* = 13), and fever (n = 5).

### Choice of antibiotic treatment

All (266/266) participants used penicillin as antibiotic first choice (in accordance with the guideline). In addition, a minority (n = 13, 5%) of GPs also used other antibiotics, including amoxicillin with clavulanic acid (*n* = 6), clarithromycin or roxithromycin (n = 6), amoxicillin (*n* = 3), and metronidazole (n = 3). For patients allergic to penicillin, 199 (75%) GPs prescribed clarithromycin or roxithromycin (which is in accordance with the guideline). Some GPs used one or more other antibiotics for those allergic to penicillin; 65 (24%) GPs used erythromycin, 13 (5%) GPs used azithromycin, and two (1%) GPs used clindamycin.

### Knowledge of and adherence to guideline

In total, 151 (57%) participants claimed to use the guideline, 77 (29%) GPs knew the guideline but reported that they did not follow it, and 38 (14%) GPs admitted that they had no knowledge of the guideline. For the group of GPs claiming to use the guideline, 17% stated that they used the guideline in all cases, 37% in 80–99% of cases, 31% in 60–79% of cases, 9 % in 40–59% of cases, and 6 % in less than 40% of cases. GPs claiming to use the guideline, used the (modified) Centor score (79%) significantly more frequently than GPs admitting to not following the guideline (45%) (*p* < 0.001, Fishers exact test), while the use of other modalities were similar between groups (streptococcal RADT: *p* = 0.52; clinical assessment: *p* = 0.12; CRP / leukocyte count: *p* = 0.32; throat swab culture: *p* = 0.34).

Two hundred three (76%) participants reported nine different reasons for not following or deviating from the guideline (Table 5).

**Table 5:** Reasons for non-adherence to the national guideline^a^ answered by 203 Danish general practitioners

Answers	N (%)
Confidence in clinical assessment	79 (39%)
Time pressure	68 (33%)
Difficulty remembering the guideline	39 (19%)
Concerns for complications	27 (13%)
Consultation is easier or faster	26 (13%)
Patient insist on antibiotic treatment	14 (7%)
Concerns for patient complaints	8 (4%)
The guideline is too simple	8 (4%)
Use of local guideline	4 (2%)
Lack of confidence in the guideline	1 (0%)

Note: Participants were asked to select one or more answers

^a^Respiratory tract infections - diagnosis and treatment 2014 (Danish Society of General Practitioners, DSAM). http://vejledninger.dsam.dk/luftvejsinfektioner/ Accessed 19 July 2018

The most common self-reported reasons for non-adherence to the guideline were confidence in the clinical assessment (39%), time pressure (33%), and difficulty remembering the guideline (19%). The reasons for non-adherence were significantly different between those claiming to use guideline versus those admitting to not following the guideline. Those claiming to use the guideline reported more frequently that they deviated from the guideline due to greater confidence in their own clinical assessment (41% vs 26%, *p* = 0.034, Fisher’s exact test) and patients insisting on antibiotic prescription (10% vs 1%, *p* = 0.019), and less commonly due to difficulty remembering the guideline (13% vs 29%, *p* = 0.010).

## Discussion

Great variations in the management of sore throat patients existed across Danish GPs. The majority of GPs were involved in the management of sore throat patients (83%), but a substantial number (62%) of GPs delegated all (17%) or selected (45%) cases to nurses or other practice staff. A recent Danish study found that sore throat patients were more likely to receive antibiotics when consulting a practice nurse compared to a doctor [5]. In light of the complexity associated with the management of sore throat patients (including inspection and obtaining tonsillar swabs) and lack of adherence to the guideline outlined in the current study, less outsourcing to non-doctors seems appropriate.

Sixty five percent of GPs calculated the modified (15%) or original (50%) Centor Score to estimate risk of GAS infection, however only three (1%) used modified Centor Score and 23 (9%) used Centor Score correctly to determine whether a streptococcal RADT should be performed (used by 96%), and to prescribe antibiotics. Hence, the majority of GPs used modalities considered important in the appointment of patients with high probability of GAS infection and benefit of antibiotic treatment [4, 12, 15, 16]. However, only 1% of GPs used the modalities in accordance with the guideline and 82% of GPs added other modalities in their calculation of bacterial etiology. Reliance on clinical judgement (67%) and biochemical infection markers (48%) were prevalent. In addition, 18% of GPs occasionally send throat swabs for bacterial culture, which in Central Region Denmark includes culturing for *Fusobacterium necrophorum*. Recent studies suggest that this anaerobe is a prevalent pathogen in complications of acute tonsillitis, and it may also play a significant role in uncomplicated sore throat [17, 18].

Fifty-seven percent of GPs claimed to use the guideline, but when asked about their management, only a minority were adherent to the guideline. This contradiction is probably based on a mixture of intentional modifications (general assessment, CRP / leukocyte count, and cultures were frequently used), misconceptions (use of streptococcal RADT in patients with low (modified) Centor scores), and lack of knowledge (non-use of (modified) Centor score).

Our findings suggest that the majority of GPs have some, but not complete confidence in the guideline. GPs weigh their clinical skills highly and seek assurance in biochemical measurements. In a study by Gröndal and colleagues, GPs considered streptococcal RADT unreliable (because pathogens other than GAS are significant) and GPs were more prone to rely on clinical assessment and CRP measurement than a streptococcal RADT [19]. In addition to a lack of confidence in the guideline, other common reasons can be categorized as lack of usability (experience that consultations lasted longer or were troublesome, time pressure, difficulty remembering the guideline etc.). Previous studies report patient demand for antibiotic therapy as a dominant factor for antibiotic overprescribing [6, 20]. In the current study, only 7 % of GPs deviated from the guideline because of patient-related pressure for antibiotic prescription.

In contrast to many other Western countries, previous studies found that Danish GPs prescribe penicillin to 86–92% of sore throat patients [5, 9, 21, 22]. We found that narrow-spectrum penicillin was first choice for all participants, thus confirming the conservative choice of antibiotics among Danish GPs. Furthermore, in accordance with the guideline, we found that the majority of GPs (75%) prescribe macrolides to patients allergic to penicillin. This poses a potential problem in patients infected with *Fusobacterium necrophorum* as this anaerobe is resistant to macrolides, and a lack of treatment may lead to an increased risk of peritonsillar abscess and Lemierre’s syndrome [2].

### Consequences of non-adherence

Multiple studies have shown that non-adherence to guidelines increases the volume of antibiotics prescribed [11, 16, 23]. Our findings suggest that the streptococcal RADT is used excessively (applied to patients with very low risk of GAS infection) potentially resulting in inappropriate antibiotic treatment of GAS-carriers with viral infection. In addition, 58% of GPs using (modified) Centor Score prescribe antibiotics without prior streptococcal RADT to patients with modified Centor Score / Centor Score 0–3, relying rather on their clinical assessment and biochemical findings. Hence, with the very low adherence rates and the prevalent use of other (diagnostic) modalities, we find a potential for reducing the use of antibiotics to Danish sore throat patients without compromising patient safety, if the guideline was better adhered to. On the other hand, the morbidity and risk of complications (especially Lemierre’s syndrome and peritonsillar abscess) in *Fusobacterium necrophorum*-positive cases is largely unexplored. These cases, as well as additional pathogens (beyond GAS) may also benefit from antibiotic treatment. The lack of high quality studies on risk factors for complications and the course of disease in sore throat patients with or without antibiotic treatment (using validated questionnaires) paves the way to skepticism towards the sore throat guideline. The guideline is both too complex and too simple for clinical use; too complex, as the majority of GPs intend to, and believe that they do largely, follow the guideline, but when tested, in fact only few adhere (in part because of difficulty recalling the guideline as outlined above), and too simple, as 82% of GPs find it rational to include additional modalities in deciding whether to prescribe antibiotics or not.

We advocate further research into the pathogenic mechanisms behind sore throat and the pathogens associated with sore throat complications. Future development of multi-pathogen RADTs may provide improved identification of patients who benefit from antibiotic treatment and reduce GPs’ concern for undertreating patients. The time is not ripe for a revision of the guidelines before the effects of antimicrobial therapy on more (than GAS) pathogens are clarified and appropriate rapid identification tests are developed. Given the low adherence to the guideline we recommend more pre- and postgraduate education for clinicians and additional health care staff to improve guideline awareness, adherence [24, 25], and recognition of its limitations.

### Strengths and limitations

This cross-sectional study gives insight into GPs’ attitudes on the management of sore throat patients with a focus on their knowledge of and adherence to the Danish national sore throat guideline by the Danish Society of General Medicine. We did not investigate the actions of GPs / practice staff and, hence, we were unable to comment on potential differences between the answers given and actual doings. This approach may bias the results, as some participants may have answered what they thought was expected rather than their actual doings. However, based on our results very few participants answered in-line with the guidelines, suggesting low guideline adherence. Moreover, our approach allowed us to gather information regarding applicability of the sore throat guideline from a large cohort of GPs rather than the limited number of participants in previous, qualitative studies [19, 26–28]. We were unable to invite approximately one third of the GPs in Central Denmark Region and only half of the invited GPs completed the questionnaire, which may bias the findings. However, age and gender distributions of participants were similar to those of all GPs in the region. Moreover, central findings are in line with previous studies from USA, Sweden and UK suggesting that the sore throat guideline is not followed [8, 26, 27] and previous Danish studies concluding that RADT is widely used [5, 6, 29] and that penicillin is the first-choice antibiotic [22, 29]. Lastly, a substantial number of GPs, outsourced some or all sore throat cases to nurses or other staff, and the management of these patients may thus differ from the answers provided by GPs.

## Conclusions

Danish GPs rarely adhere to the recommended sore throat management guideline, but rather use various combinations of additional modalities (Centor score / modified Centor score (65%), streptococcal RADT (96%), general clinical assessment (67%), biochemical parameters (48%), and throat swab cultures (18%)) in deciding whether patients require antibiotic treatment. This practice is likely to increase antibiotic prescription rates. The reasons for non-adherence to the guideline were multiple, but primarily a greater degree of confidence in own clinical judgement, time pressure and difficulties in remembering the guideline.

## Additional file

Additional file 1: Questionnaire (freely translated from Danish). (DOCX 39 kb)

## Data Availability

The datasets used and analyzed during the current study are available from the corresponding author on reasonable request. The questionnaire can be found in the appendix (see Additional file 1).

## Abbreviations

CRP: C-reactive protein
GAS: Group A streptococci
GPs: General practitioners
RADT: Streptococcal rapid antigen detection test
REDCap: Research Electronic Data Capture
