# Supplementary material for: Management of sore throat in Danish general practices
Source: BMC Fam Pract. 2019 Jun 1;20:75. doi: 10.1186/s12875-019-0970-3 (PMC6545212; doi:10.1186/s12875-019-0970-3)
Supplement: Supplementary file 1 — Questionnaire (freely translated from Danish). (DOCX 39 kb) [file 12875_2019_970_MOESM1_ESM.docx]

# Additional file 1 Questionnaire (freely translated from Danish)

**Age**

| **Sex** | Male  Female |
| --- | --- |

**Number of years in general practice (GP)?**

| **Who manages sore throat patients in your general practice?** | Doctor  Nurse  Other staff  Choose one or more answers |
| --- | --- |

| **Which guideline(s) do nurse/other staff use in the management of sore throat patients?** | I  Local guideline  Danish Society of General Medicine guideline  Other  Choose one or more answers |
| --- | --- |

**If other guideline, which one?**

| **Which modalities do you/other staff use in the management of sore throat patients?   Centor Score: Anamnestic fever**  **Abscence of cough**  **Presence of tonsillar exudates**  **Tender cervical lymph nodes   Modified Centor Score: Centor Score with the addition of age score** | Clinical assessment  Modified Centor Score  Centor Score  Streptococcal rapid antigen detection test (RADT)  C-reactive protein (CRP) and/or leukocyte count  Throat swab culture  Choose one or more answers |
| --- | --- |

**If other modalities are used, name which?**

| **(Only GPs using Centor Score)**  **Which patients are diagnosed with RADT?**  **Centor Score = CS.  Centor Score: Anamnestic fever**  **Abscence of cough**  **Presence of tonsillar exudates**  **Tender cervical lymph nodes** | All patients   CS 0   CS 1   CS 2   CS 3   CS 4   After general clinical judgement   Patients with fever  Generally unwell  Elevated CRP and/or leukocyte count  Other   I don’t use RADT  Choose one or more answers |
| --- | --- |

**If other than the above mentioned, name which ones?**

| **(Only GPs using Modified Centor Score)**  **Which patients are diagnosed with RADT?**  **Modified Centor Score = MCS.  Modified Centor Score: Anamnestic fever**  **Abscence of cough**  **Presence of tonsillar exudates**  **Tender cervical lymph nodes**  **Modified Centor Score: Centor Score with the addition of age score** | All patients   MCS 0   MCS 1   MCS 2   MCS 3   MCS 4  MCS 5   After general clinical judgement   Patients with fever  Generally unwell  Elevated CRP and/or leukocyte count  Other   I don’t use RADT  Choose one or more answers |
| --- | --- |

**If other than the above mentioned, name which ones?**

| **(Only GPs not using (modified) Centor Score)**  **Which patients are diagnosed with RADT?** | All patients   After general clinical judgement   Patients with fever  Generally unwell  Elevated CRP and/or leukocyte count  Other   I don’t use RADT  Choose one or more answers |
| --- | --- |

**If other than the above mentioned, name which ones?**

| **(Only GPs using Centor Score)**  **Which criteria results in antibiotic prescription?**  **Centor Score = CS.  Centor Score: Anamnestic fever**  **Abscence of cough**  **Presence of tonsillar exudates**  **Tender cervical lymph nodes** | No criteria, all patients   CS 0   CS 1   CS 2   CS 3   CS 4  CS 0 + positive RADT   CS 1 + positive RADT   CS 2 + positive RADT   CS 3 + positive RADT   CS 4 + positive RADT  CS 0 + generally unwell   CS 1 + generally unwell   CS 2 + generally unwell   CS 3 + generally unwell   CS 4 + generally unwell   Patients with fever  Elevated CRP and/or leukocyte count  Positive throat swab culture  Other  Choose one or more answers |
| --- | --- |

**If other than the above mentioned, name which ones?**

| **(Only GPs using modified Centor Score)**  **Which criteria results in antibiotic prescription?**  **Modified Centor Score = MCS.  Centor Score: Anamnestic fever**  **Abscence of cough**  **Presence of tonsillar exudates**  **Tender cervical lymph nodes**  **Modified Centor Score: Centor Score with the addition of age score** | No criteria, all patients   MCS 0   MCS 1   MCS 2   MCS 3   MCS 4  MCS 5  MCS 0 + positive RADT   MCS 1 + positive RADT   MCS 2 + positive RADT   MCS 3 + positive RADT   MCS 4 + positive RADT   MCS 5 + positive RADT  MCS 0 + generally unwell   MCS 1 + generally unwell   MCS 2 + generally unwell  MCS 3 + generally unwell   MCS 4 + generally unwell  MCS 5 + generally unwell   Patients with fever  Elevated CRP and/or leukocyte count  Positive throat swab culture  Other  Choose one or more answers |
| --- | --- |

**If other than the above mentioned, name which ones?**

| **(Only GPs not using (modified) Centor Score)**  **Which criteria results in antibiotic prescription?** | All patients   After general clinical judgement   Patients with fever  Generally unwell  Elevated CRP and/or leukocyte count  Positive RADT  Positive throat swab culture  Other  Choose one or more answers |
| --- | --- |

**If other than the above mentioned, name which ones?**

| **Which antibiotics are prescribed?** | Penicillin   Amoxicillin with clavulanic acid   Amoxicillin   Erythromycin   Clarithromycin / Roxithromycin   Azithromycin   Clindamycin   Ciprofloxacin   Metronidazole   Cefuroxim   Other  Choose one or more answers |
| --- | --- |

**If other than the above mentioned, name which ones?**

| **Which antibiotics are prescriped for patients allergic to penicillin?** | Erythromycin   Clarithromycin / Roxithromycin   Azithromycin   Clindamycin   Ciprofloxacin   Cefuroxim   Other  Choose one or more answers |
| --- | --- |

**If other than the above mentioned, name which ones?**

| **Do you know the Danish Society of General Medicine guideline for the management of sore throat patients?** | Yes, and I use it always or sometimes   Yes, I’ve read it but don’t use it   Yes, I’ve heard about it but don’t use it   No, never heard of it |
| --- | --- |

| **If you use the guideline, how often do you use it?** | Always   80-99%   60-80%   40-60%   20-40%   Under 20% |
| --- | --- |

| **What are the reasons for not following the guideline? (sometimes or always)** | Time pressure   Difficulty remembering the guideline   Lack of confidence in the guideline   Patient insist on antibiotic treatment   Consultation is easier or faster   Concerns for patient complaints   Concerns for complications   Confidence in clinical assessment   The guideline is too simple   Other  Choose one or more answers |
| --- | --- |

**If other than the above mentioned, name which ones?**

| **What part of the guideline is hard to remember?** | Modified Centor Score criteria   The treatment algorithm  Other  Choose one or more answers |
| --- | --- |

**If other than the above mentioned, name which ones?**

| **What part of the guideline results in your lack of confidence?** | There is other important pathogens than Group A streptococci (GAS)   RADT doesn’t give information about other  important pathogens than GAS   Penicillin isn’t the most effective antibiotic drug   Other  Choose one or more answers |
| --- | --- |

**If other than the above mentioned, name which ones?**

| **If you have any comments on the survey or on the management of sore throat patients in general, please let us know** | (free text) |
| --- | --- |
